# Social Structure and Depression in TrevorSpace

**DOI:** 10.1145/2531602.2531704

**Published:** 2014-02

**Authors:** Christopher M. Homan, Naiji Lu, Xin Tu, Megan C. Lytle, Vincent M.B. Silenzio

**Affiliations:** Department of Computer Science Rochester Institute of Technology; Department of Biostatistics University of Rochester; Department of Biostatistics University of Rochester; Department of Psychiatry University of Rochester; Department of Psychiatry University of Rochester

**Keywords:** LGBT youth, social network analysis, social media, H.5.m. Information Interfaces and Presentation (e.g. HCI): Group and Organization Interfaces

## Abstract

We discover patterns related to depression in the social graph of an online community of approximately 20,000 lesbian, gay, and bisexual, transgender, and questioning youth. With survey data on fewer than two hundred community members and the network graph of the entire community (which is completely anonymous except for the survey responses), we detected statistically significant correlations between a number of graph properties and those TrevorSpace users showing a higher likelihood of depression, according to the Patient Healthcare Questionnaire-9, a standard instrument for estimating depression. Our results suggest that those who are less depressed are more deeply integrated into the social fabric of TrevorSpace than those who are more depressed. Our techniques may apply to other hard-to-reach online communities, like gay men on Facebook, where obtaining detailed information about individuals is difficult or expensive, but obtaining the social graph is not.

## Introduction

Though the relationship between depression and social factors has been the subject of scientific inquiry since at least the late 19th century [15], our understanding of this relationship at a predictive level remains vague [21]. Prior to the explosion in popularity of online social networking it was difficult even to gather large amounts of data on social ties. Social networking sites, by contrast, are designed around the management friends lists, and thus make collecting such data much easier.

However, social support systems are complex, heterogeneous structures in which different social ties perform different roles [48]. Even though many popular social networking sites, such as Facebook and Google+, provide members some capability to organize their social ties, these capabilities are limited and there are no standard practices for using them, certainly not with respect to clarifying social support roles for the benefit of scientific research. Thus, uniform data on the *quality* or *nature* of the social ties present—what role they serve or functions they perform—are not directly available.

Nonetheless, Cohen and Wills, while calling for a better understanding of the diverse functional roles ties may play, acknowledge that viewing social ties as the undifferentiated building blocks of social structure may nonetheless capture factors that promote general psychological health [10]. In this paper, we combine network science and traditional survey methods to discover patterns in the global structural properties of a social networking site that are related to depression. We are motivated by the potential of this line of inquiry to inform the development of intelligent systems for detecting signs of depression in a social network dedicated to reducing self-harm among lesbian, gay, bisexual, trans-gender, and questioning (LGBTQ) youth. We effectively ask ourselves what the social ties can tell us, even when we regard them as completely homogenous. Doing so brings the global structure of the ties into high relief.

We compare two groups of users of TrevorSpace with respect to six network features indicative of social integration. TrevorSpace a social networking site run by The Trevor Project, the largest nonprofit organization dedicated to preventing suicide among LGBTQ youth. We relied on the social dynamics of the site to recruit the respondents. One group contains those respondents who are screened as depressed according to a standard diagnostic instrument and the other contains those who screen as nondepressed. Our results show that the non-depressed group tends to be better integrated into the Trevorspace social graph than the depressed group, and that the differences between the groups are significant.

Suicide occurs twice as frequently in LGB adolescents and young adults as in their non-LGB peers [1, 35, 38]. Stresses specific to this population (e.g., coming out, sexual orientation stigma) and lack of support (e.g., diminished social and familial support), among other factors contribute to this increased frequency [9]. Moreover, social discord is associated with more suicidal ideation among LGBT youth [27].

Compared to middle age and older LGB adults, sexual minority youth (18-29 years in age) had lower levels of perceived social support (i.e., fit and integration with their social environment) [23]. Perceived social support, connection to the LGB community, and salience with identity have all been correlated with psychological well-being [23]. As a hidden minority, LGBT individuals often compare the benefits of coming out (e.g., increased psychological well-being and improved relationships) with such risks as depression, social isolation, victimization, and giving up privilege (i.e., heterosexual and cisgender privilege) [11, 19, 31, 40].

The Internet offers individuals the capability to connect with a broader community, and facilitate identity development [19, 23, 29, 41]. For instance, online social networks provide LGBT youth with the opportunity to share coming out experiences, discuss stigma, receive support, and get involved with activism [24, 29]. More recently, LGBT youth have been using the Internet as a way to practice disclosing their sexual orientation and/or gender identity before coming out to their friends and family [24, 29].

Online communities may thus play a larger social role for LGBTQ youth than for the general population. We believe that the relative social importance of online social networks to LGBTQ youth, along with the increased prevalence of mental health issues in this population, make online communities for this population, such a TrevorSpace, ideal settings for studying the relationship between social structure and depression.

### Contributions

This paper has two main contributions. First, we show how to use the entire social graph of an online community to test the bias and confidence of a sample of the community members. Network statistics are seldom independent and identically distributed (iid), even when the population sample used to recover the network is, and so traditional statistical methods often do not apply. Much social network analysis is thus descriptive, and avoids hypothesis testing. Methods that are more statistically rigorous need to be carefully tailored to the data available and the questions asked, and it requires some explanation so that the procedures involved can be reproduced. Our tests use well established subsampling methods but are designed specifically to the data that social networking sites provide. TrevorSpace is ideal for exploring such methods as it is large enough (~20, 000 users) that scale becomes an issue, but not too large as to be unmanageable. Many online social networks are much larger than this. It would seem that our methods could be used to study subcommunities of any social networking site (note in particular that variations of snowball sampling such as respondent driven sampling are specifically designed to discover hidden communities within larger social environments [18, 20]).

The second contribution is translational [51]. To our knowledge, this is the first study to relate network structure to depression in LGB youth in an online social network. Social networking sites can be regarded as natural systems of collaborative emotional support. We use the collaborative dynamics of the site, in the form of snowball sampling, to obtain a survey that is rich in network structure, more so than what a random sample would provide.

TrevorSpace and a sister message board are monitored by a single counselor for users who show signs of emotional distress. It is very difficult for this person to keep up with the traffic on the site. We hope that our findings may ultimately lead to intelligent systems that help actively identify individuals who may be poorly socially integrated into a social environment and thus may benefit from direct intervention and thus provide assistance in situations like TrevorSpace where expert human resources are limited.

## Related Work

A rather substantial body of work exists on the use of social networking systems (SNS) to study emotion (see [5, 14, 43, 32, 24, 4, 32, 7] among many others), though comparatively little of it considers network properties. For instance, Golder and Macy study aggregate global trends in “mood,” and show, for example, that people wake up in a relatively good mood that decays as the day progresses [17]. Bollen et al. [7] show that emotionally-charged tweets are often tied to current events, such as elections and holidays. However, most of this data is, from an experimental perspective, unstructured and secondary. Obtaining data in a scientifically rigorous manner often requires interactive data collection, especially when the persons of interest may not wish to make themselves publicly known, as is the case with many LGBT youth.

A large body of literature exists on social support, and a great deal of this work depends on a much more nuanced understanding of the various, conflicting, and changing roles that social ties play [48, 39]. Such an understanding is believed to be especially important to the stress buffering functions social support can provide [10]. Obtaining such detailed information is still challenging because it usually requires the active participation of the respondents to recall detailed information on a potentially large number ties. The purpose of our work is to look precisely at what a completely unnuanced view of the social tie structure says about depression. Such views, while limited, are nevertheless believed to shed light on the degree to which individuals benefit from social integration factors [10].

De Choudhury and colleagues look at depressive disorders using a combination of SNS data and interactive surveys [12, 13]. They use Amazon Mechanical Turk to recruit and screen for depression a number of Twitter users. They then analyze a full year of Twitter posts (tweets) from before the onset of depression by those who screened positive for major depression or reported a diagnosis at some point during the previous year. In addition, they collect a full year of tweets by non-depressed participants of the crowdsourcing activity. We, by contrast, target LGBT youth on TrevorSpace and are working with much less data (to some degree our research is about patterns we can discover with a relatively small amount of sampled data paired with a complete social graph). We also look at global network properties of the complete social graph underlying our sample, while they focus on ego networks of the sampled population and on unstructured Twitter data.

Distribution-free methods such as subsampling are a well-established part of the statistics canon [33, 44]. What is novel in our work is the application of these methods to a complete social graph of the underlying population paired with traditional survey data.

## Methods

### The Location

**Figure 1:**
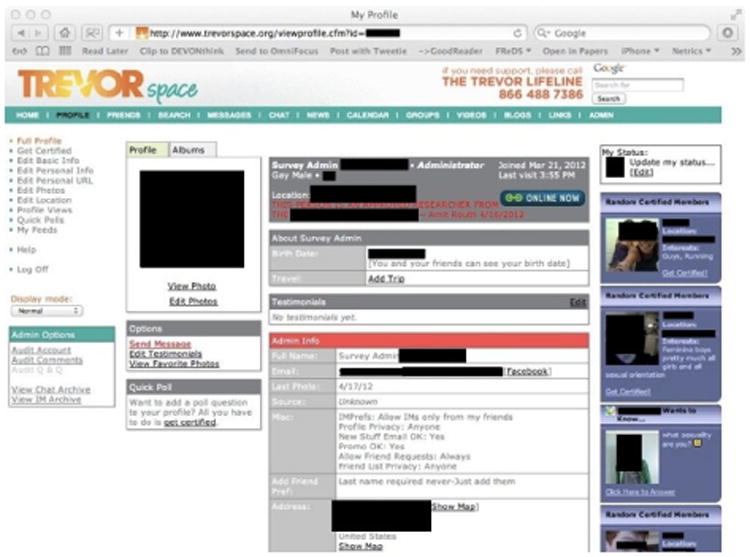
Screenshot of a TrevorSpace user's homepage.

TrevorSpace has all the salient features of a social networking site [8]: user profiles, public (to other TrevorSpace members) testimonials and comments, and publicly (again, only to other TrevorSpace members) articulated, traversable lists of friends. Additionally it provides support for members that are at risk for self harm: the site is monitored by Trevor Project administrators and links to the Trevor Helpline (a suicide prevention hotline) are posted at the top of the screen. See Figure 1. More information about the Trevor Project is available online at http://www.thetrevorproject.org/.

### The Data

The sample consisted of LGBT youth and their allies between the ages of 18 and 24 with a TrevorSpace user account that was active during the data collection period. Participation was limited to TrevorSpace users who reported suicidal ideation or a suicide attempt in the past year. Participants were excluded if they endorsed suicide ideation, did not speak English, were unable to read or complete survey materials, or had already participated in this study. Individuals who reported active suicidal ideation were referred to The Trevor Project's Lifeline for immediate care. Trevorspace users who did not complete responses to the inclusion criteria items were also excluded.

The administrative staff of TrevorSpace helped with recruitment by sending an invitation to all TrevorSpace users who were 18–24 years old. During the active recruitment phase, TrevorSpace administrators placed an advertisement on the personal homepages of TrevorSpace users. Individuals who accessed the survey site were prompted to review an information letter before they were screened for eligibility. Participants were paid $25 in an iTunes gift card for their help. This was done to help reduce sentiments of volunteerism or coercion. Once participants completed the survey, they were given the option to forward the recruitment information to their TrevorSpace friends. Thus, data was obtained via a combination of direct marketing and snowball sampling. The survey reached saturation in eight hours and was closed. Out of 270 responses, 75 individuals were excluded for not completing the PHQ9 questions in the survey or submitting duplicate responses. This resulted in a sample of 195 respondents.

Additionally, working with The Trevor Project administrators, we obtained as secondary data two snapshots of the TrevorSpace social graph, one taken March 21, the other April 20, 2012. The survey was conducted on March 31st, so it falls between the two snapshots. The March graph has 18570 and the April graph 20716 actors.

Each of these graphs was anonymized, except that we were told which survey respondent corresponds to which graph actor. This allowed us to observe the structural role of each respondent within the graph.

We performed our analysis on the union of the March and April graphs, which we call the *TrevorSpace social graph*. It has 21306 actors. Ten of the remaining 195 survey respondents do not show up in this graph (we believe they joined, then left, TrevorSpace between when the snapshots were taken), so we excluded them, leaving 185 respondents in total.

### Study Design

We based most of the survey questions (i.e., demographics, help-seeking readiness, mental health symptom history, sexual orientation stigma, and mental health stigma) on a web-based survey created by Whit-lock, Knox and colleagues [49, 50]. Additional items addressing internet and online social media use were modified from The Pew Internet and American Life Questionnaire [26].

We also embedded into the survey the PHQ9, a widely used instrument for measuring depression [34]. It has 9 questions, each of which is worth up to three points. The total score thus ranges from 0 to 27, with 0 indicating little and 27 indicating strong support for depression.

Our survey website, which was developed on Ruby on Rails with the Surveyor gem, supports complex skip patterns in the survey items [37]. Taking these into account, the average participant required between 10 and 15 minutes to complete the survey.

### Experiment Design

We create comparison groups by partitioning the respondent population into those scoring below the median PHQ9 score and those scoring no less than the median. We then compare the difference between the two groups on six different network features (described below).

96 participants have a PHQ9 score greater than or equal to the median value of 9; 89 have a lower score. A score of 9 is often taken as a cutoff for mild depression [22]. Moreover, a meta-analysis of optimal cut-off scores for depression varies; however scores between 8-11 (inclusive) tend to have similar sensitivity and specificity confidence intervals [28]. Sixteen respondents had a PHQ9 score of exactly 9. We had a choice about which group to place them in or whether to include them at all and ran our experiments each way. We only show results for the case where they are grouped with the high PHQ9 respondents; the results in the other cases were comparable if not slightly stronger. We chose to err on the conservative side.

### Network Properties

For this study, we look at six primary network features: degree, triangles, clustering coefficient, core number, and the sizes of the largest (singly) connected and biconnected component. For an overview of basic network theoretic terms, many good textbooks are available, e.g. [30, 46, 16].

A *connected component* of a graph is a maximal set of actors such that each pair is connected by a path of ties (though the actors in the pair need not themselves share a tie). A *biconnected component* is a maximal set of actors where each pair is connected by a at least two disjoint paths of ties (disjoint in the sense that neither path shares any actors, except at the end-points). In many natural and abstract graphs there is a *giant (bi)connected component* that contains most of the nodes in the graph.

The *degree* of an actor is the number of ties the actor has. This is sometimes called the *neighborhood size* and the set of actors with which the ties are formed is the *neighborhood*. We denote the neighborhood of a actor *v* in a network *G* as *N_G_*(*v*). Thus the degree is |*N_G_*(*v*)|.

A *clique* is a set of actors such that any pair of them share a tie. It is also known as a *complete graph* or an *n-clique*, where *n* is the number of actors. A *triangle* is a 3-clique. The *triangle number* of an actor is the number of triangles the actor is a member of. We denote the triangle number of actor *v* as | ∇*G* (*v*)|

The *clustering coefficient* [47] of an actor *v* is

$$|\nabla G(v)|/(\begin{array}{c} \left|N_{G}(v)\right|\\ 2 \end{array}).$$

That is, it is the number of triangles to which the actor belongs divided by the total number of triangles the actor *could* belong to, given the number of neighbors the actor has.

An *n-core* is a subgraph relative to which each member actor has a degree at least *n* [36]. The *core number* of an actor is *v* is

max{n|vis a member of somen−core}.

An important virtue of *n*-cores is that they can be computed in linear time. Most other clustering techniques require higher-order polynomial or even exponential time to compute [2].

### Bias Tests

Given the previously-discussed lack of iid sampling frames for our network statistics, we adopt a non-parametric (i.e., “distribution-free” [45]), subsampling-based approach to test the significance of our results.

**Figure 2:**
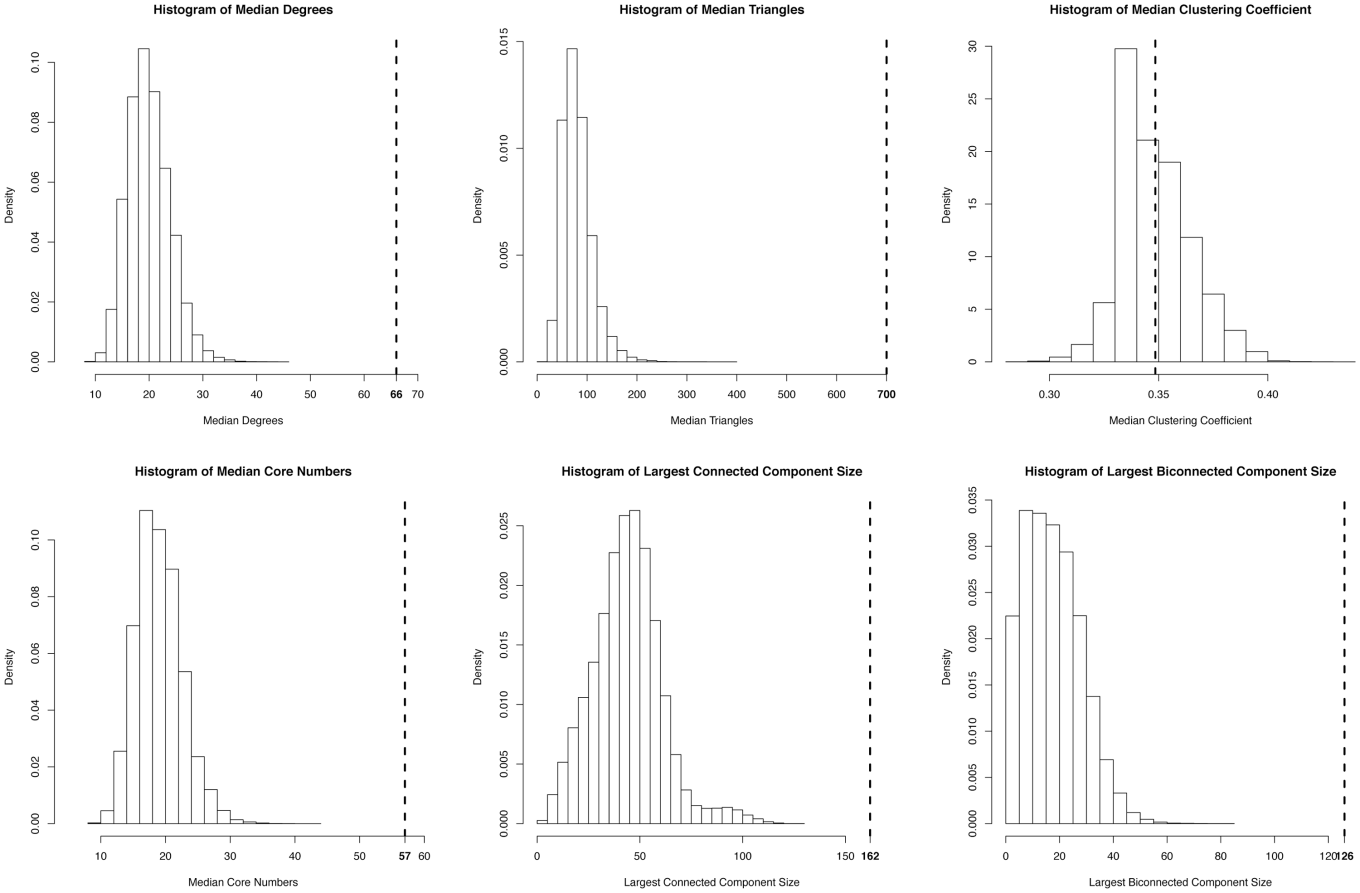
Histograms of the subsampling-based bias tests. The actual values from the survey are indicated by dotted lines. Except for clustering coefficient, the survey data appears at the right end of each histogram, indicating that the likelihood the survey data comes from a uniform random sample of the social graph is negligible.

We run two levels of tests. First, since our survey encouraged respondents to recruit their friends into the survey, we expect it to be highly unlikely that a random sample from the social graph would be more tightly interconnected, in terms of the graph features we study, than our pool of survey respondents. To test this, we take as a null hypothesis that our sample was from an independent and uniform sample without replacement from the TrevorSpace population. We simulate this by drawing without replacement a uniform random sample of 185 nodes from the TrevorSpace social graph and, for each of the six network features of interest, testing whether the the value of that feature in the sampled subgraph exceeds that of the same feature in the 185 survey respondents. We repeat this process 100,000 times and for each network feature count the number of times the comparision test is positive for that feature. This gives us an estimate of the likelihood that our survey was drawn from a random sample of the social graph. Table 1 and Figure 2 summarize these results.

### Significance Testing

**Figure 3:**
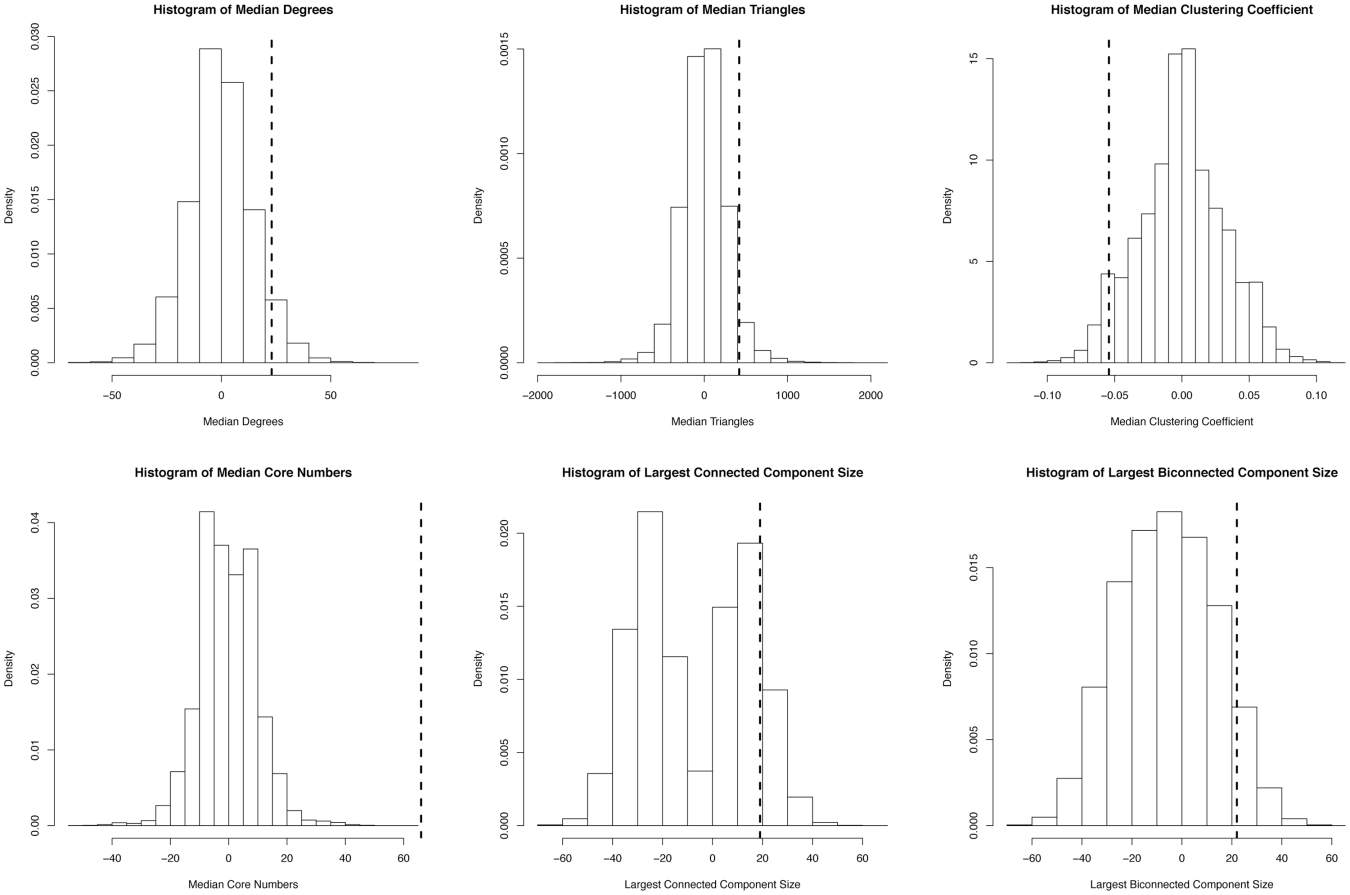
Histograms of the permutation-based significance tests of the differences between the low and high PHQ9 groups for various graph features. The actual values from the survey are indicated by dotted lines. Except for clustering coefficient, the survey results appear at the right end of each histogram, indicating that the likelihood that PHQ9 is independent of network structure is low. Clustering coefficient also appears unlikely to be independent, although in a different direction.

Second, to test the significance our results, we take as a null hypothesis that the level of depression in each of our respondents is independent the network. Then we randomly permute the respondents in the social graph (i.e., “randomly shuffle” their tie lists), keeping all other actors in the social graph fixed, and test, for each of the six network features of interest, whether the difference in that feature between the high and low groups of the permuted nodes exceeds the difference in that same feature between the high and low groups of the 185 survey respondents. We repeat this 100,000 times and for each feature count the number of times the comparison test is positive. By restricting our shuffling of actors to just the surveyed respondents, we effectively control for the effect of selection bias. Table 3 and Figure 3 summarize these results.

## Results

Figure 2 shows the results of the bias tests on the medians of degree, triangles, clustering coefficient and core number, and on the largest (bi)connected component. Table 1 summarizes these results. Table 2 shows the median values on each of these statistics for the high and low PHQ9 groups. Figure 3 and Table 3 show the significance tests on the difference between the high and low PHQ9 groups of each network feature value. In all cases the median statistics are taken from the underlying values in the social graph. The largest (bi)connected component is taken from the social graph, restricted to the group(s) of interest.

## Discussion

Figures 2 and 3 illustrate nicely the principal that network statistics do not satisfy iid assumptions, even when the population samples underlying the network samples may. By running each of the null hypothesis tests 100,000 times each, we gain fairly accurate distributions of the network statistics under uniform sampling of the actors. While some of them look normal, many do not.

The results of the bias test (Table 2) show that all of the statistics observed on the respondent data, except clustering coefficient, are extremely unlikely under the null hypothesis. It thus seems that our mixed snowball/targeted sampling process is highly biased toward samples that are much more socially integrated than a random sample. One explanation for this is that the collective dynamics of the snowball process yield a region of the social network that is structured more like a functioning community than a random sample would be. Another is that most social networking services have a large number of inactive users, and we would expect that such users would be less likely to respond to the survey (and that this would be exacerbated by the narrow time window within which the survey was conducted) and also less integrated into the network, than active users would be. There is maybe some support for both explanations.

Moving on to the high and low PHQ9 groups, Table 3 shows that all differences in network features between these groups, except for the largest connected component, are significant at the 0.1 level and triangle and core numbers are significant at the 0.05 level. Each difference statistic is significant in the direction we expect, i.e., that the low PHQ9 group appears more tightly integrated into the social network than the high PHQ9 group, except for clustering coefficient, which was significant in the opposite direction.

This would seem to indicate that although nondepressed individuals (due to their significantly higher median triangle number) have more friends who know each other than do depressed individuals, they also have proportionally more “isolated” friends who do not know each other. One interpretation is that those who are not depressed are more likely to develop friendships with people outside of their normal social circles—and are thus more socially adventurous—than those who are depressed. Related work on this matter is mixed. Like us, DeChoudhury et al. discover that depressed individuals have a higher clustering coefficient in the Twitter social graph than nondepressed people [13]. Bearman and Moody found that women who have more friends that are not friends with each other are more likely to think about suicide than those who have fewer [3].

The results of the social network analysis are consistent with previous suicide prevention research and have clinical implications. For instance, the lack of connectivity and social belongingness are associated with suicidal behaviors [42], and at times there are social costs when LGBT youth decide to come out to their friends and families. LGBT individuals may experience rejection and victimization when they disclose their sexual orientation and gender identities [11].

### Ethics and Broader Issues

This study was approved by our institutional review board (IRB). Respondents were required to read an information letter that discusses the risks and benefits of the research. Consent to participate in the study was indicated by completing the online survey and respondents could withdraw at anytime by closing their browser. Unlike secondary analysis research, we had to consider such ethical issues as privacy and potential risks. As previously mentioned, participants who endorsed suicidal ideation were excluded from this study and received immediate care from the Trevor Project.

As mentioned earlier, we are motivated by the potential of this line of inquiry to inform the development of intelligent systems for detecting signs of depression in a social network dedicated to reducing self harm among LGBT youth. It seems that our approach could also apply to other social networking sites to study similar problems. We are aware of several organizations that are on the cusp of bringing self harm reduction systems into the social networking domain. For instance, the Veterans Crisis Line^1^ provides online chat and text messaging and 911 is starting to develop the capability to accept text messages.^2^ Facebook has devoted resources and a webpage to detecting suicidal behavior among its users.^3^

The potential for the broader adoption of this approach raises a number of issues about privacy and coercion in the data gathering process, especially given the sensitive nature of both the target audience—LGBT youth—and the behavioral domain—mental health.

One way to ensure privacy in the snowball process, which we practice, is to not ask respondents to name names; rather, they find and contact all new recruits to the survey site. This is an explicit part of respondent-driven sampling (RDS), a widely-used variant of snowball sampling [18]. Only those who choose to step forward and be surveyed are thus known to us. Our IRB was especially careful about how we did this; we were not allowed to observe who recruited whom, even after the data was anonymized. TrevorSpace is particularly careful about protecting the identities of its users. In general, however, a social networking site can monitor any such survey activity in the site, and thus gain information about membership in a hidden community or other sensitive information. Ways to minimize such privacy risks include using site resources, such as APIs, as little as possible and informing participants of the risks.

These methods also raise concerns about coercion, by both the administrators of the site and the snowball process. A related, though less nefarious, worry is that volunteerism can bias the sample in undesirable ways. We did not receive any complaints of coercion. By paying participants we hoped to help mitigate these effects by providing a clear reward for participating. Also, site administrators remained detached and impersonal in targeting site members.

### Limitations

Given our desire to obtain a relatively well-connected sample, we did not randomly select participants. Moreover, the sample was collected over a very short period of time (one day). Thus it must be regarded as a convenience sample. There are few if any fully random surveys on LGBT youth.

The population of interest—recently suicidal LGBT youth—is very difficult to access and we were not able to obtain a comparison group (with a comparative network structure) via anything resembling a fully experimental design. This is a very specific group, one that is marginalized and relatively unhappy, and they were recruited from a social venue that is tailored to their needs. The degree to which our results apply to other populations is not yet clear.

A basic goal in social network research is to distinguish influence from selection when correlations are observed among closely related actors. For instance, we observed a slight anticorrelation in the PHQ9 scores of TrevorSpace friends. Is this because, say, depressed people have a slight tendency to not select each other as friends, or does having depressed friends cause one to become less depressed? We do not have the data to answer this question. Our primary concern here is to establish correlations between network features and depression, given a rather small population sample and the complete social graph of the underlying population.

### Changes over time

It is known that levels of depression can fluctuate over time and even vary throughout the day [6, 16]. Social networks also change; for instance, in the month-long window between the two network snapshots of Trevorspace we used to construct the social graph, the network lost 590 users and gained 2,736. Kumar, Novak and Tomkins show that the edge density of online social networks fluctuates dramatically over time [25].

It is unclear whether one's level of social integration fluctuates dynamically with mood. We currently have survey data from one point in time only. We do have multiple snapshots of the social graph, and we did some exploration into whether depression in the survey data relates to changes in the social graph, but have yet to discover any convincing evidence.

## Conclusion and Future Work

This paper discovered network signatures for depression in an social networking service. We partitioned the participants of a survey on TrevorSpace, an SNS dedicated to reducing suicide and self harm among LGBTQ, youth into two groups, based on their score on a standard instrument for diagnosing depression. We believe our techniques of combining detailed, respondent-driven survey data with easy-to-obtain but low fidelity social tie data may apply to other hard-to-reach online communities on social networking sites.

The are a number of future directions to go with this work. An obvious goal is to build and assess models that use network structure to assess or predict who in a social network is or might become depressed, and then use this information to stage successful interventions. The techniques we develop here have shed important light on which network features may be most useful in this regard.

In obtaining network data, there seems to be a fundamental tradeoff between samples that are unbiased and those, such as from peer referral processes, that provide rich network structure. Is it possible to control snowball dynamics in a way that balances this tradeoff? For instance, RDS purports to provide a less biased sample than other peer referral methods, through a combination of limiting the number of people any one respondent can recruit and sophisticated estimators [18]. Could the features of a social networking site be used to somehow interactively and differentially regulate the recruitment process to make it more representative of, alternately, the population or the network?

Another next step would be to monitor the level of connectivity and depression LGBT youth have from when they first engage in a supportive network until they choose to leave. This information would help determine whether or not users were reaching out for support when they are depressed and exiting when they have improved psychological well-being. These results could then inform the monitoring of crisis and suicide prevention networks and the training of expert monitors. Such a study also could help tease apart the cause/effect dynamics between social structure and suicide.

Finally, we hope to combine the methods described here with those that uncover more detailed information about the functions and roles specific social ties play. Can the features of social networking sites help with this? We would expect that, at a minimum, the friends lists most sites provide would help the recall process, e.g., by carefully prompting the respondent with information gleaned from the site.

## Figures and Tables

**Table 1:** The size of the medians of degree, triangle number, clustering coefficient and core number, calculated from the social graph, and of the giant (bi)connected component of the subgraph of respondents, along the results of the bias test.

Feature	Value	P-value
Size	185	n/a
Giant Con. Comp.	162	< 10^−5^
Giant Bicon. Comp.	126	< 10^−5^
Med. Degree	66	< 10^−5^
Med. Triangles	700	< 10^−5^
Med. Clustering	0.3485	0.45
Med. Core Number	57	< 10^−5^

**Table 2:** The sizes of the giant connected, and biconnected components, and the median degree, triangle number, clustering coefficient and core number, calculated from the social graph, of the low and high PHQ9 respondent group significance tests.

Feature	PHQ9 < 9	PHQ9 ≥ 9
Size	89	96
Giant Con. Comp.	68	49
Giant Bicon. Comp.	52	30
Med. Degree	78	55
Med. Triangles	967	547.5
Med. Clustering	0.316722	0.3709409
Med. Core Number	66	48.5

**Table 3:** The significance tests (“p-value”) of the differences (“ Δ”) of the statistics from Table 2, calculated in the social graph, of the low and high PHQ9 respondents.

Feature	A	P-value
Giant Con. Comp.	19	0.15
Giant Bicon. Comp.	22	0.086
Med. Degree	23	0.059
Med. Triangles	419.5	0.049
Med. Clustering	−0.05	0.059
Med. Core Number	17.5	0.039

## Footnotes

1 http://www.veteranscrisisline.net

2 http://www.fcc.gov/text-to-911

3 https://www.facebook.com/help/contact/305410456169423
